# Primary Small Cell Carcinoma of Liver: A Rare Tumor

**DOI:** 10.4021/gr2010.06.215w

**Published:** 2010-07-20

**Authors:** Lileswar Kaman, Javid Iqbal, Mahander Pall, Amanjit Bal

**Affiliations:** a Department of General Surgery, Postgraduate Institute of Medical Education and Research, Chandigarh-160012, India; b Department of Histopathology, Postgraduate Institute of Medical Education and Research, Chandigarh-160012, India

**Keywords:** Primary small cell carcinoma liver, Liver tumor, Bisectionectomy of liver

## Abstract

Primary small cell carcinoma of the liver is very rare tumor. Till date only 12 cases have been reported in the English literature. We are reporting a case of primary small cell carcinoma of the liver in a female patient. She had 13 cm x 7 cm tumor in the right lobe of liver and fine needle aspiration cytology revealed features of small cell carcinoma. After ruling primary from elsewhere, patient underwent central bisectionectomy of the liver and histopathology confirmed the diagnosis of primary small cell carcinoma of the liver. On immunohistochemistry examination, the tumor was positive for Neuron-specific enolase and synaptophysin but negative for Thyroid transcription factor 1 and Hep-Par 1. Here we discuss the clinical course and treatment of primary small cell carcinoma of the liver in our case and review the literature.

## Introduction

Majority of small cell carcinomas (SCC) are located in the lungs, which account for 25% of lung carcinomas [1, 2]. About 2% - 4% of small cell carcinomas have been reported from extrapulmonary organs, including esophagus, thymus, stomach, pancreas and cervix [1-3]. These are diagnosed as extrapulmonary small cell carcinomas (EPSCC). Almost half of the EPSCC are localized in the gastrointestinal tract [2-3]. The occurrence of EPSCC in other organs is considered to be rare [1-3]. Primary small cell carcinoma (PSSC) of the liver is very rare entity and only 12 cases have been reported worldwide. We report a case of 40-year-old female patient with primary small cell carcinoma of the liver.

## Case Report

**Figure 1:**
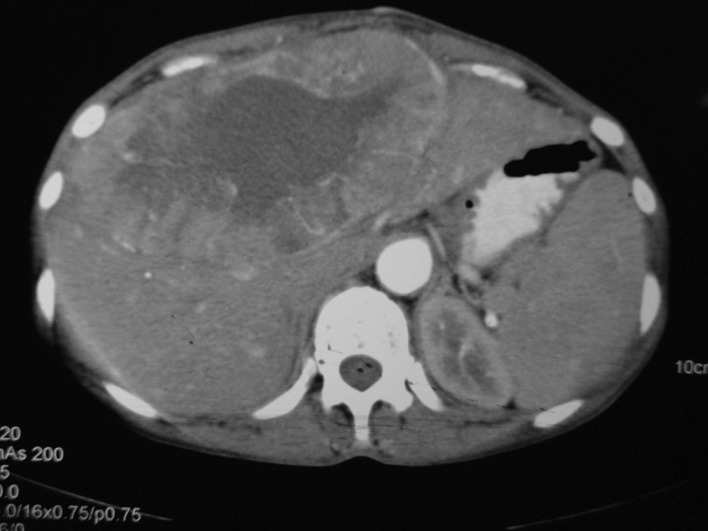
Contrast enhanced computed tomography (CECT) abdomen revealed 13.2 x 13.5 x 7.3 cm well defined mass lesion involving segment IV, V and VIII of liver. There was rim enhancement on arterial phase and no contrast retention on venous phase.

**Figure 2:**
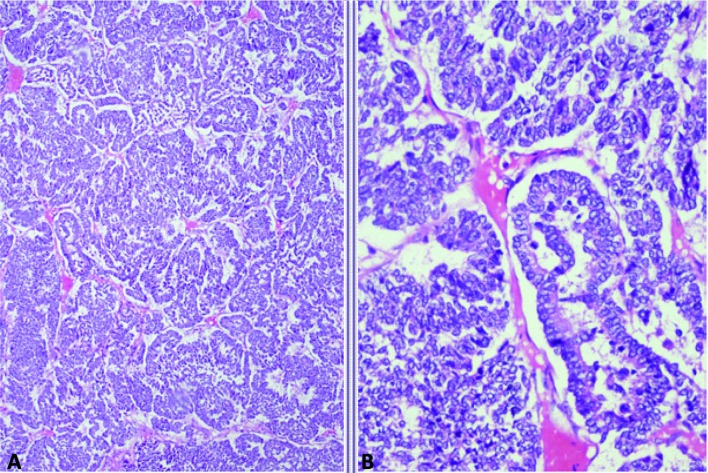
Photomicrograph showing A: Organoid and trabecular pattern of tumor cells separated by thin fibrovascular septa; B: Tumor cells with finely granular nuclear chromatin and scanty cytoplasm (Haematoxylin and eosin, x 100, x 400).

**Figure 3:**
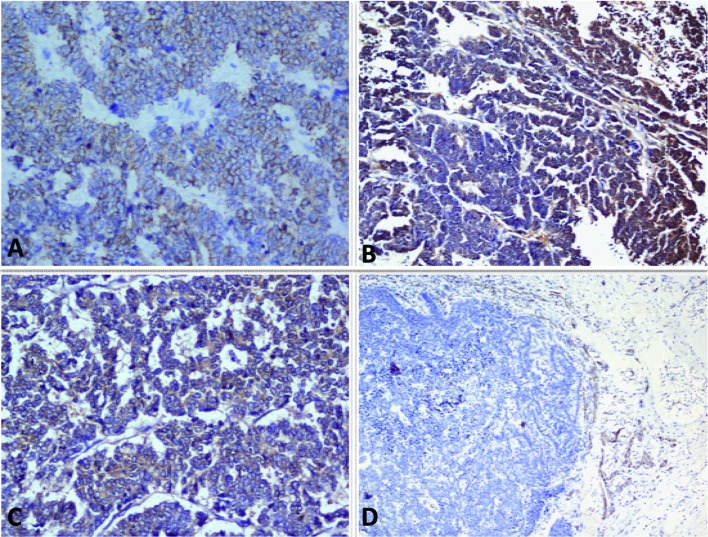
A-D: Photomicrograph showing A: Pancytokeratin positivity; B: Neuron specific enolase positivity; C: Synaptophysin positivity and D: Hep Par-1 negativity in tumor cells (Immunostains, x 200).

A 40 years old female patient presented with pain upper abdomen, mild to moderate in intensity for 40 days and lump upper abdomen for 10 days. She had history of loss of weight and appetite, but there was no history of cough, fever, jaundice or oral contraceptive intake. On examination, patient was thin built, afebrile, non icteric and hemodynamically stable. Chest and cardiovascular examination were within normal limits. Hepatomegaly was present with an 8 cm x 7 cm lump in the epigastric region. There was no ascites and rectal examination was within normal limit. On laboratory examination, the hemoglobin was 9.8 gm%, serum bilirubin was 0.24 gm%, AST and ALT were 63/53 IU, alkaline phosphate was 572 IU, albumin was 3.5 gm%, PTI was 100%, Alpha Feto Protein was 2.11 ng/dl and CEA level was 1.0ng/ml, which were all within the normal limits. Contrast enhanced computed tomography (CECT) abdomen revealed 13.2 x 13.5 x 7.3 cm well defined mass lesion involving segment IV, V and VIII of liver (Fig. 1). There was rim enhancement on arterial phase and no contrast retention on venous phase. Lesion was compressing the right and left branches of portal vein and there was no ascites. Tumor was involving 58.37 cm^2^ (38.68%) of the total liver volume. Fine needle aspiration cytology (FNAC) showed features of small cell carcinoma the liver. Upper and lower gastrointestinal endoscopy were normal. Chest x-ray, sputum cytology, chest CECT and bronchoscopy were normal. Positron Emission Tomography (PET) scan revealed 14 x 10 cm mass lesion in liver with metabolic active disease at periphery and central necrosis. No other hypermetabolic lesions elsewhere in body were seen. Patient underwent laparotomy and central bisectionectomy for the tumor arising from segment IV, V and VIII of liver. Histology showed tumor arranged in lobules, nests and trabecular pattern (Fig. 2). Cells were mildly pleomorphic with hyperchromatic nucleus, scanty cytoplasm and many mitotic figures. Features were suggestive of primary small cell carcinoma of the liver. Portal lymph nodes showed metastatic disease. On immunohistochemistry, tumor was positive for neuron-specific enolase and synaptophysin but negative for thyroid transcription factor 1, hep-Par 1 and carcinoembryonic antigen (Fig. 3). Patient recovered well in postoperative period, and was discharged on postoperative day 7. Now patient is on follow-up and undergoing combined chemotherapy.

## Discussion

Since its initial description by Duguid and Kennedy in 1930, extrapulmonary small cell carcinoma (EPSCC) is recognized as a clinicopathological entity distinct from small cell carcinoma of the lung [4-5]. EPSCC have been reported to occur in 0.1% - 0.4% of all malignancies [1-3, 6-7]. It has been recognized as a distinct clinical entity and has been reported in several organs other than the lung over the past 30 years. Primary locations include the head, neck, salivary glands, thyroid, larynx, trachea, thymus, pleura, esophagus, stomach, intestines, rectum, pancreas, gall bladder, cervix, uterus, breast, prostate, urinary bladder, and skin [1-3]. In order to distinguish EPSCC from metastatic pulmonary small cell carcinomas, patient should have normal chest radiograph, computed tomography (CT) scan of the chest, sputum cytology, and negative bronchoscopy or PET scan. Primary small cell carcinoma of the liver (PSCCL) is very rare and only 12 cases have been described in the literature until now (Table 1). Zanconati et al [8] reported 3 cases, one patient was treated by mass resection but the others received no therapy. The clinical progression was rapid and death ensued between 1 and 5 months after diagnosis. In the two cases reported by Sengoz et al [9], one patient who received chemotherapy survived for 13 months and the other survived for 67 months after hemihepatectomy. Kim et al [10] reported a case in which segmentectomy of the liver and adjuvant chemotherapy were performed, the patient survived with no signs of recurrence for at least 4 months. In other 5 reported cases, 2 patients received combined chemotherapy [11, 12], and two underwent resection [13, 14] and one received no treatment [2]. Choi et al [12] reported that primary small cell cancer of liver expresses c-kit, a stem cell marker of the liver. Morikawa et al [15] reported a case of primary small cell liver which was treated with chemotherapy with good outcome. In our patient tumor was arising from segment IV, V and VIII of liver and underwent central bisectionectomy followed by combined chemotherapy and is doing well. The patients of primary small cell cancer of liver, who underwent radical operation and chemotherapy, had better outcome. The regimens of chemotherapy consisted of platinum-based combination therapy. Because of the small number of cases reported till date, it is difficult to make any definite recommendation regarding the best possible treatment for primary small cell cancer of liver. But knowing the aggressive course of the tumor behavior, it is better to go for radical surgery and followed by combination chemotherapy.

**Table 1:** Clinicopathological and Immunohistochemical Features of Patients With Primary Small Cell Carcinoma of the Liver

Author	Age /Sex	Symptom	Size (cm)	Stage of Disease	AFP (ng/ml)	Immunohistochemical Staining	Treatment
Ryu et al [11]	56/M	RUQ pain, general weakness	8 cm	Advanced	3.24	(+) CD56, C-kit, SYN; (-) TTF-1	CT (cisplatin, etoposide, irinotecan)
Kim et al [10]	53/M	Palpable mass	12 cm	Advanced	2.94	(+) CD56, NSE, C-kit, SYN, mixed CK, EMA; (-) CK7, 8, 19, 20, AFP, CEA, hepatocyte, vimentin, desmin, TTF-1	Segmentectomy and CT (cisplatin, etoposide)
Zanconati et al [8]	56/M	Abdominal discomfort	5 cm	Limited	> 200	(+) AE1/AE3, CK8, 18, 19, NSE, AFP, ERY-1; (-) S-100 protein, CEA	Partial hepatectomy
Zanconati et al [8]	69/M	DM, weight loss	10 cm	Advanced	-	(+) AE1/AE3, CK 8, 18, 19; (+/-) NSE, CHR; (-) S-100 protein, CEA	-
Zanconati et al [8]	89/M	Jaundice	6 cm	Advanced	150	(+) AE1/AE3, CK 8, 18, 19, AFP, NSE; (-) CHR, S-100 protein, CEA	-
Kim et al [13]	67/M	Abdominal discomfort	12 cm	Advanced	-	(+) SYN, CD56, C-kit; (-) CK, CEA, AFP	CT (cisplatin, epirubicin)
Sengoz et al [9]	73/F	-		Advanced	-		Right hepatectomy
Sengoz et al [9]	66/M	-		-	-		CT (Cisplatin)
Kim et al [2]		-		Advanced		(+) CHR, SYN	-
Choi et al [12]	82/F	Abdominal discomfort	6.7 cm	Advanced	3.4	(+) CD56, NSE, SYN, CHR,TTF-1, C-kit; (-) Antihepatocyte, AFP,vimentin, desmin, CK7, 19, 20, CEA, S-100 protein	Segmentectomy of liver and CT (etoposide)
Morikawa et al [15]	77/M	General fatigue and breathlessness	10 cm	Advanced	27	(+)AE1/AE5, CAM5.2; (-) NSE, desmin, vimentin, CEA	CT (cisplatin, etoposide)
Yang-Qing Huang et al [14]	34/M	Incidental detection		-	-		Right hepatectomy and segment I excision and TACE for recurrance
Index case	40/F	Pain and lump abdomen	13.5 cm	Advanced	2.1	(+) NSE, SYN; (-) TTF1, hep-par 1, CEA	Central bisectionectomy and CT (cisplatin, etoposide)

RUQ, Right upper quadrant; DM, Diabetes mellitus; CT, Chemotherapy; AFP, Alpha-fetoprotein; CEA, Carcinoembryonic antigen; CHR, Chromogranin; CK, Cytokeratin; EMA, Epithelial membrane antigen; NSE, Neuron-specific enolase; SYN, Synaptophysin; TTF-1, Thyroid transcription factor 1.
